# Is Dental Caries a Modern Disease?

**Published:** 1883-12

**Authors:** 


### IS DENTAL CARIES A MODERN DISEASE?

For many years dentists have been informing their patients that caries of the teeth is owing to modern methods of living, especially to deprivation of the necessary lime salts by the vicious modes of the proparation of our food. Many practitioners have been in the custom of prescribing lime in various farms, as a prophylactic against decay. It has been repeatedly asserted in our societies, journals, and elsewhere, that the nations whose food was rich in phosphatic material had good teeth, and *vice versa.* Now the real facts of the case are, that a careful examination of race teeth actually and unmistakably disproves these assertions.

The attention of dentists seems now turned toward the investigation of this subject, and it may be hoped that the result will be an increase in knowledge concerning a point that should have been unmistakably established long ago. We publish in this number letters from valued correspondents, which indicate the contradictory views heretofore held, and show the great need there is for a more careful examination of the craniums of the extinct races, where such are obtainable. Our Rome correspondent promises an investigation of the skulls preserved there, and residents of other countries or localities affording opportunities for anthropological study are earnestly invited to make examinations as thorough as circumstances will permit, and to transmit the record to this journal for publication.

The editor of the INDEPENDENT PRACTITIONER is not the first in this field, by any means. Dr. J. J. R. Patrick, of Belleville, Illinois, has made some careful investigations, the results of which have been published, and Dr. Rollins, of Boston, we are informed, has examined the skulls at Cambridge, though we are not aware that he has published anything upon the subject. There are also others to whom credit should be given for original research, and we hope the list will be largely extended in the coming year.

